# Hemoglobin expression reduces aerobic bioconversion of guaiacol in *Pseudomonas putida* despite non-limiting oxygen

**DOI:** 10.1186/s13568-026-02089-3

**Published:** 2026-06-30

**Authors:** Fredrik Lund, Matilda Dellson, Nélida Leiva Eriksson, Marie Gorwa-Grauslund, Magnus Carlquist

**Affiliations:** 1 Division of Biotechnology and Applied Microbiology, Department of Process and Life Science Engineering, Lund University, Lund, SE-221 00 Sweden; 2 Novonesis, Gammel Venlighedsvej 14, Hoersholm, 2970 Denmark

**Keywords:** *Pseudomonas putida*, Hemoglobin, Oxygen, Lignin, Aromatics catabolism

## Abstract

Efficient lignin valorization relies on oxidative enzymatic reactions, making oxygen availability a critical determinant of process performance. *Pseudomonas putida*, an obligate aerobe and promising host for valorization of aromatic compounds, experiences reduced growth and conversion rates under oxygen limitation. Here, two plant hemoglobin genes originating from *Arabidopsis thaliana* (*AHb2*) and *Beta vulgaris* (*BvHb2*) were expressed, and the resulting strains were evaluated for growth on glucose and vanillin, and for whole-cell bioconversion of guaiacol to muconic acid. Expression of hemoglobin showed a tendency toward improved growth on glucose, but not on vanillin, in shake flask cultivations. In contrast, expression of *AHb2* in a strain co-expressing *gcoAB* for conversion of guaiacol to cis, cis-muconic acid resulted in reduced growth and respiration under well-controlled bioreactor conditions, despite non-limiting oxygen. Furthermore, lower conversion rates of guaiacol to cis, cis-muconic acid were observed without affecting product yield, indicating that hemoglobin expression influences how oxygen is utilized within the cell rather than improving its availability for bioconversion.

**Supplementary Information:**

The online version contains supplementary material available at https://doi.org/10.1186/s13568-026-02089-3.

## Introduction

*Pseudomonas putida*, a gram-negative, organoheterotrophic soil bacterium, has gained interest as a host for industrial and environmental biotechnology (Martin-Pascual et al., 2021; Nikel and de Lorenzo 2018) due to its robust and versatile metabolism, rapid growth, tolerance to physicochemical stress, and availability of genetic engineering tools (Martínez-García and de Lorenzo 2024). Consequently, *P. putida* has been the basis of advanced synthetic biology and metabolic engineering efforts aimed at producing valuable chemicals or degrading environmental pollutants (de Lorenzo et al. 2024; Nikel and de Lorenzo 2018). *P. putida* can catabolize a wide range of structurally diverse aromatic compounds into convergent bioproducts (García-Hidalgo et al. 2019; Kohlstedt et al. 2018, 2022; Salvachúa et al. 2018), which, along with its innate tolerance to aromatics (Salvachúa et al. 2018), has promoted its choice as the workhorse organism for biological valorization of lignin (Beckham et al. 2016).

The catabolic pathways for assimilation of aromatic compounds can be tuned to produce specific chemicals of high value (Abdelaziz et al. 2016; Weiland et al. 2022). *P. putida* has notably been engineered to convert lignin-derived compounds into muconic acid (Almqvist et al. 2021; Kohlstedt et al. 2018, 2022; Salvachúa et al. 2018; Werner et al. 2023), beta-ketoadipate (Werner et al. 2023) and adipic acid (Lund and Gorwa-Grauslund 2024), i.e., dicarboxylic acids that are used in applications such as the production of polyamides. Oxygen plays a major role in the catabolism of aromatic compounds due to the involvement of molecular oxygen in the mechanism of several monooxygenase and dioxygenase enzymes present in the catabolic pathways (Abdelaziz et al. 2016; Weiland et al. 2022). However, scaling-up aerobic microbial processes typically leads to oxygen limitation due to the low solubility of oxygen (Kampers et al. 2021). Continuous supply of air or pure oxygen can be used, although it comes at a considerable capital cost (Humbird et al. 2017). Even with sufficient supply of oxygen, local anoxic environments may also exist due to mixing limitations (Lara et al. 2006). Therefore, genetic engineering strategies to improve the performance of *P. putida* at low oxygen levels should be explored to aid in the scale-up of microbial bioprocesses. Improved performance at low oxygen levels could also enable coupling of aerobic catabolism with anaerobic and/or oxygen-sensitive enzyme activities in the same *P. putida* strain.

*P. putida* uses oxygen as the terminal electron acceptor in the electron transport chain (ETC). Implementing anaerobic growth in *P. putida* has been the topic of several engineering strategies (Kampers et al. 2019; Nikel and de Lorenzo 2013; Steen et al. 2013) and, although anaerobic fermentation and survival have been demonstrated, growth remains to be achieved and is predicted to require more than 50 genetic changes (Kampers et al. 2021). A simpler strategy to reduce the need for oxygen may be to employ an oxygen carrier in the form of a hemoglobin protein.

Hemoglobin encompasses a family of proteins that are involved in oxygen binding and transport, that are found across all kingdoms of life. Hemoglobin contains a heme b prosthetic group within a globular protein structure connected covalently to a histidine side chain. The heme group contains an iron atom that is involved in the binding of oxygen. The role of hemoglobin as an oxygen transporter in vertebrates is well-established, but its role in plants and prokaryotes appears to be more diverse and not limited to interactions with oxygen (Hoy and Hargrove 2008).

The first identified bacterial hemoglobin was isolated from *Vitreoscilla stercoraria* (VHb) as a homodimeric species (Wakabayashi et al. 1986). VHb is a well-studied hemoglobin that has been cloned in several species with the objective of improving respiration, growth and product formation under oxygen-limited conditions. It was first introduced in *Escherichia coli*, where it was shown to improve microaerobic cell growth and enhance productivity of recombinant proteins (Khosla and Bailey 1988; Khosravi et al. 1990; Stark et al. 2015).

The present study explores the impact of expressing hemoglobin as an oxygen binding protein on the performance of *P. putida*. Two types of hemoglobin genes (*AHb2*, and *BvHb2*) with different oxygen affinity were expressed and evaluated for their effect on microbial growth in M9 medium with glucose, and vanillin, and for whole-cell bioconversion of guaiacol to muconic acid.

## Materials and methods

### Strains and media

Chemicals were purchased from Sigma Aldrich (St. Louis, MO, USA) and were of analytical grade. *E. coli* DH5α, used for subcloning, plasmid storage and propagation, was cultured at 37 °C in lysogeny broth (LB) (peptone (10 g/L), yeast extract (5 g/L) NaCl (10 g/L) (Sambrook 2001). *P. putida* KT2440 (Bagdasarian et al. 1981) was cultured at 30 °C in either LB or M9 medium (12.8 g/L Na_2_HPO_4_, 3 g/L KH_2_PO_4_, 0.5 g/L NaCl, 1 g/L NH_4_Cl, 2 mM MgSO_4_, 0.1 mM CaCl_2_) (Sambrook 2001) supplemented with a trace element solution (500 mg/L EDTA disodium dihydrate, 200 mg/L FeSO_4_ ⋅ 7 H_2_O, 10 mg/L ZnSO_4_ ⋅ 7 H_2_O, 3 mg/L MnCl_2_ ⋅ 4 H_2_O, 30 mg/L H_3_BO_3_, 20 mg/L CoCl_2_ ⋅ 6 H_2_O, 1 mg/L CuCl_2_ ⋅ 2 H_2_O or CuSO_4_ ⋅ 5 H_2_O, 2 mg/L NiCl_2_ ⋅ 6 H_2_O, 3 mg/L Na_2_MoO_4_ ⋅ 2 H_2_O) (Pfennig and Lippert 1966). Glucose (10 g/L (55 mM), vanillin (5 mM), guaiacol (5 mM) and 5-aminolevulinic acid (5-ALA, 0.3 mM) were added as mentioned in the text. Streptomycin (100 µg/mL) was added for plasmid maintenance.

A list of all strains and plasmids used in the study can be found in Table 1.

**Table 1:** List of strains and plasmids used in the study

Strain	Genotype	Reference
*P. putida* KT2440	Parental strain	DSM 6125, ATCC 47,054
TMB HV 001	*P. putida* KT2440 Δ*catBC*	(Almqvist et al. 2021)
TMB FL 028	*P. putida* KT2440 Δ*catBC* pFL027	(Lund and Gorwa-Grauslund 2024)
TMB FL 030	*P. putida* KT2440 Δ*catBC* pFL030	This study
TMB FL 034	*P. putida* KT2440 Δ*catBC* pFL034	This study
TMB FL 036	*P. putida* KT2440 Δ*catBC* pFL008	(Lund and Gorwa-Grauslund 2024)
TMB FL 038	*P. putida* KT2440 Δ*catBC* pFL038	This study
TMB FL 048	*P. putida* KT2440 pFL030	This study
TMB FL 049	*P. putida* KT2440 pFL008	This study

Plasmid	Relevant features	Reference
pBSK-FP1-NAP_AtHb2	T7, *AHb2*	This study
pBSK-FP1-NAP_BvHb2	T7, *BvHb2*	This study
pFL008	RK2 oriV, p14g, T0, Sm^R^	(Lund and Gorwa-Grauslund 2024)
pFL027	RK2 oriV, p14g, *P450Rrh*,* FrdRrh*, T0, Sm^R^	(Lund and Gorwa-Grauslund 2024)
pFL030	RK2 oriV, p14g, *AHb2*, T0, Sm^R^	This study
pFL034	RK2 oriV, p14g, *BvHb2*, T0, Sm^R^	This study
pFL038	RK2 oriV, p14g, *AHb2*, *P450Rrh*,* FrdRrh*, T0, Sm^R^	This study

### Molecular biology

Standard molecular cloning techniques were used (Sambrook 2001). FastDigest restriction enzymes and T4 DNA ligase were purchased from ThermoFisher Scientific (Thermo Fisher Scientific, Waltham, MA, USA). Phusion High Fidelity DNA Polymerase from ThermoFisher Scientific (Thermo Fisher Scientific, Waltham, MA, USA) was used for cloning PCR reactions. DreamTaq DNA polymerase from ThermoFisher Scientific was used for colony PCR. Plasmids were purified using the GeneJet Plasmid miniprep kit from ThermoFisher Scientific (Thermo Fisher Scientific, Waltham, MA, USA). PCR products were purified using either the GeneJet PCR purification kit or the GeneJet Gel Extraction kit from ThermoFisher Scientific (Thermo Fisher Scientific, Waltham, MA, USA).

Oligonucleotides were synthesized by Eurofins Genomics (Ebersberg, Germany). Synthetic genes codon-optimized for *E. coli* (Suppl. Table S1) were synthesized by Epoch Biolabs (USA). All constructed plasmids were verified by Whole Plasmid sequencing performed by Eurofins Genomics (Ebersberg, Germany).

### Plasmid construction

pFL030 was constructed by amplifying the *AHb2* gene from plasmid pBSK-FP1-NAP_AtHb2 using primers FL_AtHb_fw_BamHI and FL_AtHb_rev_RBS_KpnI (Suppl. Table S2). The amplicon was purified, digested with *Bam*HI and *Kpn*I and ligated into pFL008 that had been digested with *Bam*HI and *Kpn*I.

pFL034 was constructed by amplifying the *BvHb2* gene from plasmid pBSK-FP1-NAP_BvHb2 using primers FL_BvHb_rev_BamHI and FL_BvHb_fw_RBS_KpnI (Suppl. Table S2). The amplicon was purified, digested with *Bam*HI and *Kpn*I and ligated into pFL008 that had been digested with *Bam*HI and *Kpn*I.

The plasmid for co-expression of AHb2 and GcoAB encoding genes (pFL038) was constructed by PCR amplifying the *AHb2* gene using primers FL_AtHb_RBS_XmaJI and FL_AtHb_rev_SacI (Suppl. Table S2). The PCR product was purified, digested with *Xma*JI and *Sac*I and ligated into pFL027 that had been digested with *Xma*JI and *Sac*I.

### Transformation

*P. putida* was transformed by electroporation as previously described (Lund and Gorwa-Grauslund 2024; Manfrao-Netto et al. 2021). Briefly, *P. putida* was grown in LB to mid-exponential phase (Optical Density at 620 nm; OD_620_ ~0.8–1.5) after which it was washed several times in ice cold 300 mM sucrose, lowering the volume each time until a final volume of 500 µL. 100–500 ng of plasmid was added to 100 µL aliquots. The aliquots were then transferred to an electroporation cuvette (0.2 cm, Bio-Rad). Electroporation was performed using a Bio-Rad Genepulser (Bio-Rad Laboratories, Inc, Hercules, CA, USA) with settings 2.5 kV, 200 Ω and 25 µFD. Cells were plated on LB plates with streptomycin after 1 h recovery in LB medium. Positive clones were verified by colony PCR.

### Shake flask cultivations

Shake flask cultivations were performed in M9 medium in 250 mL Erlenmeyer flasks (with or without baffles as specified) with a working volume corresponding to 10% of the nominal flask volume (25 mL). Cultures were incubated at 30 °C either under agitation at 180 rpm or under static conditions in an incubator (New Brunswick™ Innova^®^ 43 Incubator Shaker, Eppendorf^®^, Hamburg, Germany). Cultures were inoculated to an initial optical density at 620 nm (OD₆₂₀) of 0.1. Samples (1 mL) were withdrawn at regular intervals for measurement of OD₆₂₀ and analysis of extracellular metabolites by high-performance liquid chromatography (HPLC). Two or three independent biological replicates were performed for each strain and condition as specified in the text.

### Bioreactor cultivations

Precultures of *P. putida* strains TMBFL028 and TMBFL038 were prepared in M9 medium in 500 mL shake flasks by inoculating single colonies from freshly streaked agar plates and incubating in a for 24–36 h. Bioreactor cultivations were performed in M9 medium supplemented with 10 g L⁻¹ glucose, 0.3 mM 5-aminolevulinic acid (ALA), and 5 mM guaiacol, using 2‑L stirred‑tank bioreactors (Infors HT, Bottmingen, Switzerland) operated at a working volume of 1 L. The bioreactors were controlled using eve^®^ software (Infors HT, Switzerland).

Cultivations were conducted at 30 °C with an agitation speed of 500 rpm and an aeration rate of 0.5 vvm. The pH was maintained at 7.0 by automated addition of 3 M KOH. Dissolved oxygen (DO) was monitored using a DO probe (Infors HT), and the exhaust gas composition (O₂ and CO₂) was analyzed using an off‑gas analyzer (BlueSens, Germany).

Samples were withdrawn at defined time points for determination of optical density, cell dry weight (CDW), and extracellular concentrations of glucose, guaiacol, and muconic acid, which were quantified by high‑performance liquid chromatography (HPLC).

Three independent biological replicates were performed for each strain and condition.

### Biomass determination

Biomass concentration was monitored by measuring optical density at 620 nm (OD₆₂₀) using a spectrophotometer (U-1100 spectrophotometer, Hitachi, Japan). Cell dry weight (CDW) was determined gravimetrically. Culture samples of defined volume were harvested by centrifugation at 10,000 × g for 5 min, washed once with deionized water, and dried at 105 °C for 24 h.

### Chromatographic analysis

Aromatic compounds (vanillin, vanillic acid, guaiacol) and muconic acid were analyzed using reverse-phase chromatography on a Waters HPLC system (Waters Binary HPLC pump 1525, UV/Visible detector 2489, Autosampler 2707) (Waters Corporation, Milford, USA) equipped with a Select C18 column (150 mm x 4.6). An isocratic method (Manfrao-Netto et al. 2021) of 65% trifluoroacetic acid (0.1%) in MilliQ H20 (A) and 35% acetonitrile (B) was used at a flow rate of 1 mL/min with a run time of 10 min per sample. Analytes were detected at a wavelength of 281 nm. Standards of each compound between 0.05 and 3 mM were used to make calibration curves. Samples were diluted with MQ H_2_O to be within the range of the standard curve.

Glucose was analyzed on a Bio-Rad Aminex HPX-87 H column (300 × 7.8 mm, 8 μm) (Hercules, CA) coupled to a Shimadzu RID-6 A RI detector (Kyoto, Japan) with 5 mM H_2_SO_4_ as the mobile phase at a flow rate of 0.6 mL/min and the temperature of the column was kept at 60 °C (Osiro et al. 2019).

### Calculations

Gas exchange rates were determined from off-gas analysis using inlet and outlet gas compositions together with the recorded gas flow rate. The carbon dioxide evolution rate (CER, mmol·L⁻¹·h⁻¹) and oxygen uptake rate (OUR, mmol·L⁻¹·h⁻¹) were calculated as volumetric rates, and the respiratory quotient (RQ, dimensionless) was defined as the ratio of CER to OUR (RQ = CER/OUR). All rate-based parameters were determined during strain-specific exponential growth phases, identified by stable CER and OUR signals. The volumetric oxygen transfer coefficient (kₗa, h⁻¹) was estimated from off-gas measurements under bioreactor conditions by relating oxygen transfer rates to the difference between the dissolved oxygen concentration and its saturation value. The maximum oxygen transfer rate (OTRₘₐₓ, mmol·L⁻¹·h⁻¹) was calculated as OTRₘₐₓ = kₗa·C*, where C* represents the dissolved oxygen concentration at saturation (mmol·L⁻¹). The specific growth rate (µ, h⁻¹) was calculated from OD₆₂₀ measurements by linear regression of the natural logarithm of optical density versus time during exponential growth. Biomass concentrations (X, g·L⁻¹) used for normalization of rates were determined as cell dry weight (CDW). The specific glucose consumption rate (qGlucose, g·g⁻¹·h⁻¹) was calculated as the rate of glucose depletion normalized to the average biomass concentration in the same interval. Specific respiration rates were defined as qO₂ (mmol·g⁻¹·h⁻¹) = OUR/X and qCO₂ (mmol·g⁻¹·h⁻¹) = CER/X, where X represents the average biomass concentration (g·L⁻¹) in the selected time window. Specific substrate and product conversion rates were calculated as qGuaiacol (mmol·g⁻¹·h⁻¹) and qMA (mmol·g⁻¹·h⁻¹), respectively. Biomass yield on substrate was calculated as Yx/s (g·g⁻¹), and product yield on substrate as Yp/s (mol·mol⁻¹). Welch’s t-test, assuming a two-tailed distribution and unequal variance between samples, was used to determine statistical significance at α = 0.05. Statistical analysis was limited to key parameters directly related to growth and product formation.

### Bioinformatic analysis

Multiple sequence alignment was performed using the Clustal Omega software, accessed via the EMBL-EBI Job Dispatcher (Madeira et al. 2024).

Phylogenetic analysis was performed with the IQ-Tree software (Trifinopoulos et al. 2016) using standard settings (1000 bootstrap alignments, 1000 maximum iterations, 0.99 minimum correlation coefficient). The phylogenetic tree was visualized using the software Interactive Tree of Life (Letunic and Bork 2024).

Pairwise sequence alignment was performed using the EMBOSS Needle software accessed via the EMBL-EBI Job Dispatcher (Madeira et al. 2024).

### Spectrophotometric quantification of hemoglobin in cell extract

Protein extraction was performed by collecting 44 mL cell suspension after 15 h of cultivation, which was then treated with B-PER™ Bacterial Protein Extraction Reagent (Thermo Fisher Scientific, Waltham, MA, USA) according to instructions provided by the manufacturer. Samples were sparged with CO during and after cell lysis to minimize oxidation of the proteins.

Hemoglobin was quantified as described previously (Cooper et al. 2024). Optical spectra were taken using a Cary 50 spectrophotometer (Agilent) within the range of 200–700 nm. The deoxyHb form was made by the addition of a slight excess of sodium dithionite to the Hb sample. Then this was bubbled with CO to produce carbonmonoxyHb which represents the functional hemoglobin protein. The amount of hemoglobin was calculated based on the maximum absorbance within wavelength range 414–418 nm. Welch’s T test, assuming two-tailed distribution and unequal variance between samples, was used to determine if values were significantly different when α = 0.05.

## Results

### Choice of suitable hemoglobin candidates

Two plant hemoglobin (Hb) genes (*AHb2*,* BvHb2*, Table 2) were chosen to be evaluated as oxygen carriers in *P. putida.* They varied slightly in terms of kinetic properties and oxygen affinity (Table 2). The genome of *Arabidopsis thaliana* contains at least two non-symbiotic hemoglobin genes, *AHb1* and *AHb2*, whose products are thought to have distinct biological functions: while Hb1 acts as a NO scavenger, Hb2 is proposed to act as an oxygen carrier (Spyrakis et al. 2011). The physiological function of *Beta vulgaris* Hb2 (encoded by *BvHb2*) is not fully understood, but it has been proposed to have a role in the regulation of intracellular NO levels; it has also been hypothesized to maintain ATP levels during vernalization and flowering (Leiva-Eriksson et al. 2014; Leiva Eriksson et al. 2019). Among the two candidates chosen, AHb2 has a slightly higher oxygen affinity.

**Table 2:** Hemoglobin candidates and their origin, kinetic parameters, and oxygen affinity

Hemoglobin origin	Gene name	Physiological role	Kinetics	Reference
*Arabidopsis thaliana*	*AHb2*	Oxygen carrier	K_on_ = 614 × 10^6^ M^− 1^ K_off_ = 0.14 s^− 1^	(Leiva Eriksson et al. 2019; Spyrakis et al. 2011)
*Beta vulgaris*	*BvHb2*	NO regulation, ATP level maintenance	K_on_ = 766 × 10^6^ M^− 1^ K_off_=0.094 s^− 1^	(Leiva Eriksson et al. 2019)

**Fig. 1:**
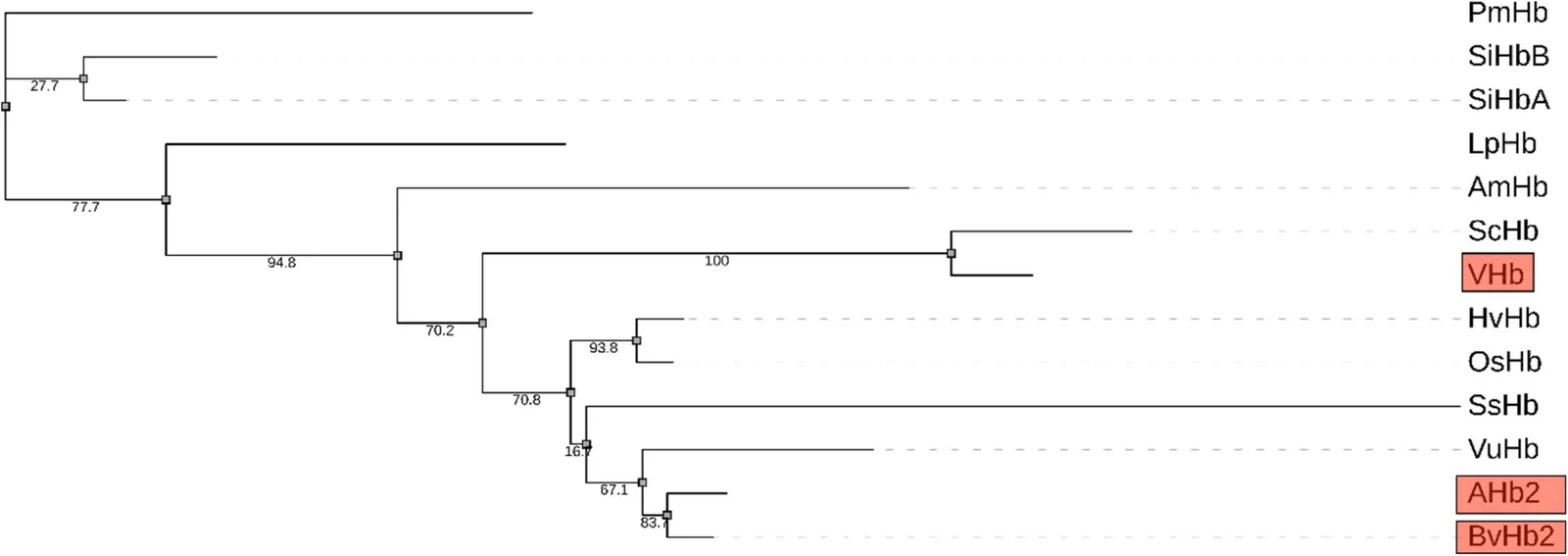
Phylogenetic tree of hemoglobin proteins that have been produced microbially along with the chosen candidates. The two plant hemoglobins and the commonly used hemoglobin VHb are marked in red. The sequences used can be found in Table S3 in the supplementary information

The two hemoglobins were compared phylogenetically to 10 other hemoglobin proteins that have been microbially produced (Fig. 1). As seen in Fig. 1, AHb2 and BvHb2, clustered together, which suggests homology. Indeed, the two hemoglobins of plant origin share a sequence identity of 72.8%. They also clustered distinctly away from the well-characterized hemoglobin, VHb, with a sequence identity of 20.8% (AHb2) and 23.4% (BvHb2), respectively.

### Over-expression of plant hemoglobin genes and impact on cell growth in shake flasks

**Fig. 2:**
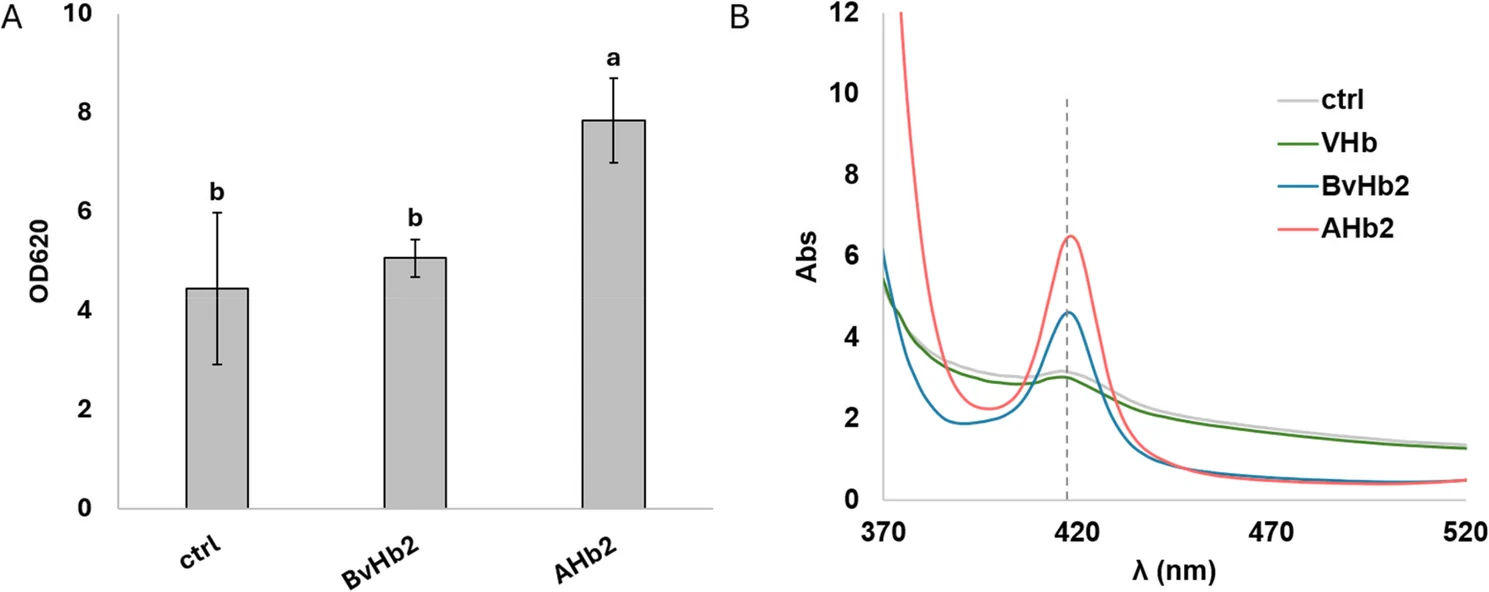
Growth and hemoglobin expression in *P. putida* during shake flask cultivation. **A** Final optical density at 620 nm (OD₆₂₀) of the control strain (TMBFL036) and strains expressing *BvHb2* (TMBFL034) and *AHb2* (TMBFL030) cultivated in M9 medium. Data represent the mean of three biological replicates, and error bars indicate standard deviation. Statistical significance between groups is indicated where *p* < 0.05. **B** Absorbance spectra of cell extracts. Hemoglobin-specific absorbance maxima are observed at 414–418 nm (dotted vertical line). Data shown correspond to one biological replicate

The effect of adding the chosen hemoglobin genes on *P. putida* cell growth was evaluated in aerobic batch cultivation in shake flasks. Hemoglobin genes were expressed on multicopy plasmids under the control of the strong, constitutive promoter p14g (Zobel et al. 2015). The expression products of *AHb2* and *BvHb2* were confirmed by the presence of a 17 kDa band on an SDS-PAGE gel (Supplementary Fig. 1), and by a distinct red color of the cell lysate (Supplementary Fig. 2). An attempt to express *vgb* from *Vitreoscilla stercoraria* was also made, however, no visible band at 17 kDa was observed and the cell lysate did not display a reddish hue, indicating that the hemoglobin gene was not functionally expressed with the used expression system. Spectrophotometric analysis of cell extracts confirmed the presence of functionally folded hemoglobin in strains expressing *AHb2* and *BvHb2*, with estimated concentrations of 0.92 ± 0.30 mg·L⁻¹ and 0.61 ± 0.23 mg·L⁻¹, respectively (Fig. 2B; Supplementary Fig. 3A). In contrast, extracts from the *vgb*-expressing strain showed spectra indistinguishable from the control strain, further supporting the absence of functional protein expression.

Biomass formation of strains expressing Hb was compared to the control strain in aerobic batch cultivation in M9 medium in shake flasks. Growth was higher for the strain expressing *AHb2*, with a significantly higher final OD compared to both the control strain without hemoglobin and the strain expressing *BvHb2 *(*p* < 0.05) (Fig. 2A). BvHb2 showed a tendency toward improved growth compared to the control strain, although this effect was not statistically significant (*p* = 0.56). It was speculated that Hb expression could further improve growth under reduced aeration. Strains producing BvHb2 and AHb2 were therefore cultivated under conditions of reduced aeration. VHb was excluded from these experiments since it was not produced in *P. putida*. As expected, low aeration under static growth conditions led to poor growth across all strains, with increased lag phase, lower growth rate, and reduced final OD, indicating that the cultures were oxygen-limited. Under these conditions, a tendency toward improved growth was observed for strains expressing both hemoglobin genes compared to the control strain lacking Hb (Supplementary Fig. S4), although these trends could not be confirmed statistically. Taken together, these observations suggest that AHb2, and possibly also BvHb2, may enhance growth under certain conditions, although controlled bioreactor cultivations provide a more suitable context for resolving these effects.

### Effect of hemoglobin on native oxygen-dependent reactions

The next step was to assess whether hemoglobin expression influences native oxygen-dependent reactions required for growth. In *P. putida*, vanillin assimilation involves several oxygen-dependent enzymatic steps (Supplementary Fig. S5A). Specifically, vanillate O-demethylase (VanAB), a Rieske-type monooxygenase, converts vanillic acid to protocatechuic acid in an oxygen-dependent reaction, while protocatechuate 3,4-dioxygenase catalyzes the subsequent oxygen-dependent ring-cleavage step.

During growth on vanillin, vanillin is rapidly converted to vanillic acid, after which assimilation proceeds and biomass formation is initiated (Ravi et al. 2017). As these downstream steps depend on molecular oxygen, growth on vanillin provides a system to evaluate the effect of hemoglobin expression on native oxygen-dependent metabolism.

**Fig. 3:**
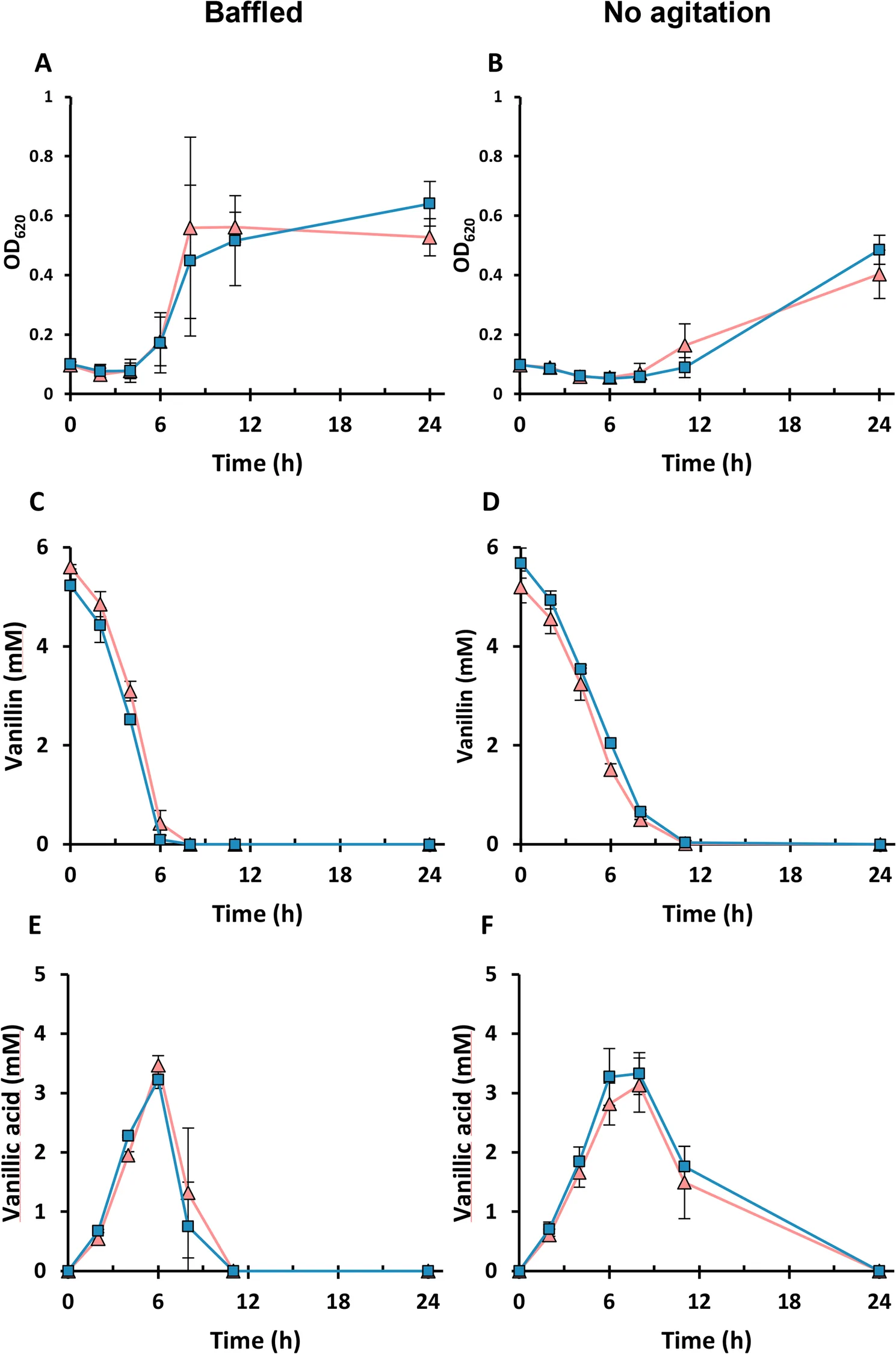
Growth and vanillin consumption of *P. putida* expressing *AHb2* (TMBLF048, red triangles) or carrying an empty vector (TMBFL049, blue squares). Cultivations were performed in M9 supplemented with 5 mM of vanillin. Two different conditions were used: baffled shake flask at 180 rpm (left column, **A**, **C**, **E**) and baffled shake flask at 0 rpm (right column, **B**, **D**, **F**)). Cell growth (OD_620_) is presented in panels A and B; vanillin concentration (mM) in panels C and D; and vanillic acid concentration (mM) in panels E and F. Data represent the mean of three biological replicates, and error bars indicate standard deviation

Growth on vanillin was therefore evaluated for the strain expressing *AHb2* (TMBFL048) and compared to an empty vector control (TMBFL049). To investigate whether aeration could influence the outcome, cultivations were performed under both shaking and static conditions. Vanillin consumption and formation of vanillic acid showed similar profiles for both strains, and complete conversion of vanillin occurred prior to the onset of biomass formation. No significant differences in growth rate or biomass formation were observed between the strains or between aeration conditions, indicating that hemoglobin expression does not enhance vanillin assimilation under the tested conditions (Fig. 3).

### Impact of hemoglobin expression on whole-cell bioconversion of guaiacol to muconic acid

The effects of AHb2 on the whole-cell bioconversion of guaiaol to muconic acid was assessed in a strain expressing the guaiacol demethylase system (GcoAB) from *Rhodococcus rhodochrous* (García-Hidalgo et al. 2019). This NADH-dependent system requires molecular oxygen in the enzyme mechanism of action where guaiacol is converted to catechol, generating formaldehyde as a byproduct (García-Hidalgo et al. 2019). Catechol is further converted to muconic acid (MA) by *P. putida* through the action of a native enzyme, CatA, that also requires molecular oxygen (Supplementary Fig. S5B). Further deletion of *catBC* genes enables muconic acid accumulation (Almqvist et al. 2021; Kohlstedt et al. 2018).

To enable quantitative assessment of oxygen consumption and its relationship to cellular metabolism, the experiments were conducted under well-controlled conditions in bench scale bioreactors. This allowed for real-time measurement of dissolved oxygen as well as off-gas composition (CO₂ and O₂), for quantification of carbon dioxide evolution rate (CER) and oxygen uptake rate (OUR).

Whole-cell bioconversion experiments were performed in M9 medium supplemented with the heme precursor 5’-aminolevulinic acid (5-ALA). ALA supplementation significantly improved the expression of *AHb2*, and hemoglobin production was also visually evident from the distinct red coloration of the culture (Supplementary Fig. S3B).

**Fig. 4:**
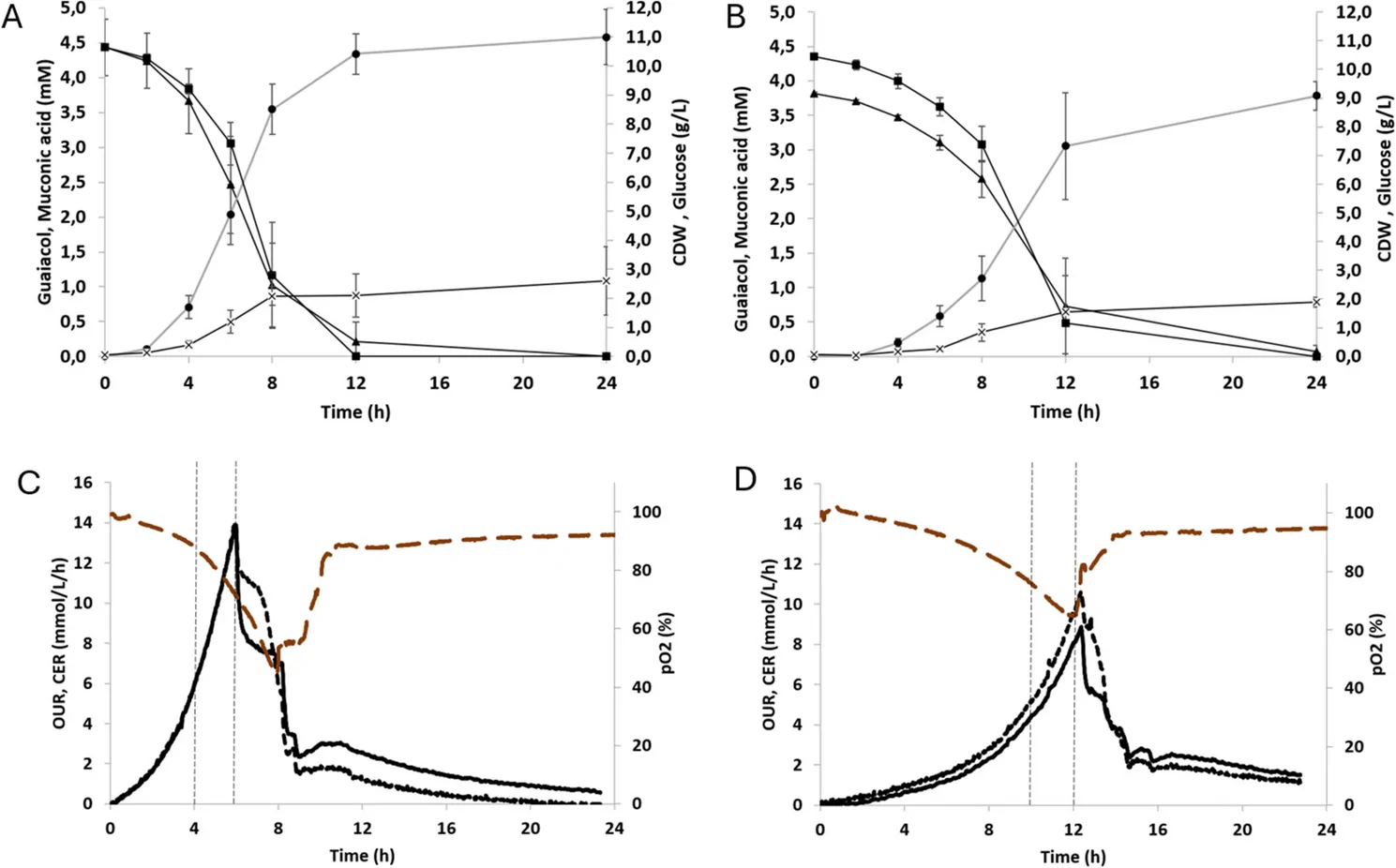
Whole-cell bioconversion of guaiacol to cis, cis-muconic acid and respiratory activity in bioreactor cultivations of *P. putida*. **A**–**B**) Time profiles of guaiacol consumption, muconic acid (MA) production, biomass formation (CDW), and glucose consumption for strains lacking hemoglobin (TMBFL028) (**A**) and expressing *AHb2* (TMBFL038) (**B**). The left axis shows concentrations of guaiacol (filled triangles) and muconic acid (filled circles) in mM, while the right axis shows cell dry weight (crosses, g·L⁻¹) and glucose (filled squares, g·L⁻¹). Data represent the mean of biological triplicates, and error bars indicate standard deviation. **C**–**D** Carbon dioxide evolution rate (CER, solid line), oxygen uptake rate (OUR, short dashed line), and dissolved oxygen (pO₂, long brown dashed line, % air saturation) for TMBFL028 (**C**) and TMBFL038 (**D**), shown for representative cultivations. Dashed vertical lines indicate the time window used for calculation of the respiratory quotient (RQ), corresponding to the exponential growth phase for each strain

In bioreactor cultivations, TMBFL028 and TMBFL038 exhibited distinct differences in growth, substrate consumption, and product formation dynamics (Fig. 4A–B). TMBFL028 followed a typical batch profile, with a short lag phase (2–4 h) followed by rapid exponential growth between 4 and 8 h, during which CDW increased to approximately 2.1 g·L⁻¹. This was accompanied by rapid glucose consumption, with depletion occurring by 12 h. Guaiacol was consumed in parallel, decreasing from approximately 4.6 mM to near depletion by 12 h, while muconic acid accumulated quickly, reaching approximately 5.0 mM at 12 h.

In contrast, TMBFL038 displayed delayed and more gradual dynamics, with a longer lag phase (4–6 h) and exponential growth occurring between 6 and 12 h. Biomass formation remained lower throughout the cultivation, reaching approximately 1.6 g·L⁻¹ at 12 h and 1.9 g·L⁻¹ at 24 h. Glucose consumption was correspondingly slower, with depletion occurring between 12 and 24 h. Guaiacol conversion proceeded more gradually, and muconic acid formation lagged behind that of TMBFL028, reaching approximately 3.6 mM at 12 h.

These differences were reflected in the physiological parameters measured during the pseudo–steady-state phase (Table 3; Fig. 4C–D). TMBFL028 exhibited faster growth (µ = 0.40 h⁻¹) compared to TMBFL038 (µ = 0.25 h⁻¹), together with higher volumetric CO₂ production, while oxygen uptake rates were of similar magnitude in both strains. Although volumetric oxygen uptake rates were similar within experimental variation, the lower biomass-specific oxygen uptake rates (qO₂) in TMBFL038 indicate reduced cellular metabolic activity, consistent with its lower growth rate. As a consequence, TMBFL038 showed a lower respiratory quotient (RQ ≈ 0.84) than the control strain (RQ ≈ 0.96), indicating a shift in the balance between CO₂ formation and oxygen consumption.

**Table 3:** Physiological and bioconversion parameters during the pseudo–steady-state phase in bioreactor cultivations of *P. putida*. Specific rates and yields were calculated over the time intervals indicated in Fig. 4, corresponding to the exponential growth phase of each strain. Values represent the mean ± standard deviation of biological triplicates

Parameter	TMBFL028	TMBFL038
Specific growth rate (µ) (h^− 1^)	0.40 ± 0.01^a^	0.25 ± 0.01^b^
CER (mmol L^− 1^ h^− 1^)	7.53 ± 1.68	5.96 ± 0.01
OUR (mmol L^− 1^ h^− 1^)	7.86 ± 1.64	7.08 ± 0.01
RQ (CER/OUR)	0.96 ± 0.05	0.84 ± 0.00
qCO2 (mmol g^− 1^ h^− 1^)	10.2 ± 2.4	5.1 ± 0.8
qO2 (mmol g^− 1^ h^− 1^)	10.5 ± 2.2	6.1 ± 0.9
qGlucose (g g^− 1^ h^− 1^)	2.41 ± 0.15	3.79 ± 1.57
qGuaiacol (mmol g^− 1^ h^− 1^)	0.80 ± 0.13	0.38 ± 0.07
qMA (mmol g^− 1^ h^− 1^)	0.89 ± 0.18^a^	0.40 ± 0.08^b^
Yx/s (g g^− 1^)	0.17 ± 0.02	0.08 ± 0.03
Yp/s (mol mol^− 1^)	1.11 ± 0.05	1.04 ± 0.01

Statistical comparisons (Welch’s t-test, *p* < 0.05) were performed for selected parameters (µ, qMA, qO₂ and qCO₂); superscript letters indicate significant differences where applicable

*CER* Carbon dioxide evolution rate, *OUR* Oxygen uptake rate, *RQ* Respiratory quotient (CER/OUR), *qCO₂* and *qO₂* specific CO₂ production and O₂ uptake rates, *qGlucose* and *qGuaiacol* specific substrate consumption rates, *qP* specific muconic acid production rate, *Yx*/*s* biomass yield on glucose, *Yp*/*s* muconic acid yield on guaiacol

When normalized to biomass, TMBFL028 displayed higher specific CO₂ production and oxygen uptake rates (qCO₂ = 10 mmol·g⁻¹·h⁻¹ and qO₂ = 10 mmol·g⁻¹·h⁻¹), whereas both rates were markedly reduced in TMBFL038 (qCO₂ ≈ 5 and qO₂ ≈ 6 mmol·g⁻¹·h⁻¹). In contrast, the *AHb2*-expressing strain showed higher glucose uptake rates but lower biomass yields (Yx/s ≈ 0.08 g·g⁻¹ compared to 0.17 g·g⁻¹ in TMBFL028), indicating reduced biomass formation efficiency. Consistently, both guaiacol uptake and muconic acid production rates were lower in TMBFL038, although the overall conversion efficiency of guaiacol to muconic acid remained close to stoichiometric in both strains (Yp/s ≈ 1 mol·mol⁻¹).

The observed differences in respiration were not associated with oxygen limitation, as dissolved oxygen (pO₂) remained well above limiting levels in both cultivations (Fig. 4C–D), reaching minimum values of approximately 45% for TMBFL028 and 60% for TMBFL038. The decrease in dissolved oxygen reflects transient limitations in oxygen transfer capacity during periods of peak demand. The maximum OTR of the system was 7.5 mmol L⁻¹ h⁻¹, which is in the same range as the measured OUR of 7.86 ± 1.64 and 7.08 ± 0.01 mmol L⁻¹ h⁻¹ for TMBFL028 and TMBFL038, respectively. The decrease in pO₂ coincided with periods of highest respiratory activity, occurring earlier and more sharply in TMBFL028 and later and more gradually in TMBFL038. Consistent with this, TMBFL038 exhibited reduced growth and lower specific respiration rates, resulting in a physiological behavior that resembles oxygen-limited growth.

## Discussion

The goal of this study was to investigate whether expression of hemoglobin, an oxygen-binding and transporting protein, in *P. putida* could sustain respiration and aromatic conversion under reduced oxygen availability. Two recombinant plant hemoglobins from *A. thaliana* (AHb2) and *B. vulgaris* (BvHb2) were successfully produced individually. However, under well-controlled aerobic batch cultivation in bioreactors, growth and respiration rates were reduced, accompanied by a decrease in respiratory quotient, leading to a phenotype resembling oxygen-limited conditions despite non-limiting dissolved oxygen. Taken together, these observations suggest that the primary effect of hemoglobin expression is a shift in respiratory metabolism rather than improved oxygen delivery. Furthermore, hemoglobin expression negatively affected strain performance during aromatic bioconversion, resulting in reduced productivity of muconic acid from guaiacol under these conditions.

The study also revealed no consistent difference in growth between the strain expressing *AHb2* and the strain expressing *BvHb2* across all tested conditions, even though the hemoglobins have distinct kinetic profiles and oxygen affinity. Although neither hemoglobin has previously been introduced in bacteria for in vivo studies, the expression of other hemoglobin genes has shown that differences in growth in microaerobic conditions can arise depending on the hemoglobin (Bollinger et al. 2001). It is possible that the plasmid-based overexpression used in our study results in an expression level so high that it masks differences between the two hemoglobins. Tuning the expression, for instance by using promoters of different strengths or inducible expression systems, may reveal differences in growth based on which hemoglobin is used.

The observed negative effect of expression of Hb on oxygen dependent bioconversions of aromatics is different from previous reports, where enhanced growth, increased yields of recombinant proteins and improved productivity upon addition of a recombinant hemoglobin are reported, especially during oxygen limitation (Bollinger et al. 2001; Frey et al. 2011; Kallio et al. 1994; Khosla et al. 1990; Kim et al. 2005). The most commonly used hemoglobin in a metabolic engineering context is VHb from *V. stercoraria* (Stark et al. 2012, 2015). Numerous studies have shown its positive impact on growth and productivity, mainly in *E. coli*, but also in other species (Hofmann et al. 2009; Mirończuk et al. 2019; Ruohonen et al. 2006). In *P. putida* specifically, few studies have been conducted, the results of which are inconclusive. Gong et al., reported that expression of *Vitreoscilla* Hb (*vgb*) in *P. putida* improved growth under oxygen-limitation; however, a strain harboring nine heterologous genes (one of which was *vgb*) was compared to the wildtype KT2440 strain, making it difficult to definitely connect the improvement in growth to the expression of *vgb* (Gong et al. 2016). In another study, Ouyang et al. reported that expression of *vgb* in *P. putida* KT2442 improved the activity of a toluene dioxygenase catalyzing the conversion of toluene, benzene and chlorobenzene into their corresponding diols by as much as 160%; however, no effect on growth was observed (Ouyang et al. 2007). Another study investigated the effect of *vgb* on PHA production in *P. putida*. Similar to our findings, they reported that expression of *vgb* had a negative impact on growth and PHA production (Oliveira et al. 2020). They also found that expression of *vgb* did not influence the oxygen usage of *P. putida*, contrary to what has been reported in *E. coli*,* Enterobacter aerogenes*,* P. aeruginosa* and *Pichia pastoris.* VHb is distinctly different from the hemoglobins tested in our study. It has low sequence identity and exhibits different kinetics and oxygen affinity.

The reason for the negative impact of AHb2 on the production of muconic acid from guaiacol remains to be elucidated. Both AHb2 and the cytochrome P450 of GcoAB contain a heme group. As a consequence, overexpression of both genes may lead to a problematic competition for the heme supply. The genome of *P. putida* contains only a few genes encoding heme-containing proteins (Bleem et al. 2022). Therefore, the native heme biosynthesis machinery may not be efficient enough to supply sufficient heme for the proper folding of both heterologous heme-proteins and native heme-proteins. However, supplementation with 5-aminolevulinic acid (ALA) was used to increase heme availability and supported efficient expression of the hemoglobin, while the reduced growth and bioconversion persisted under these conditions. This suggests that the observed effects cannot be explained by heme limitation alone. Insufficient heme supply has been reported as a bottleneck for cytochrome P450 biocatalysis in *E. coli* (Hu et al. 2023) and hemoglobin production in *Bacillus subtilis* (Wang et al. 2025). In *E. coli*, several strategies to improve heme supply have been reported including addition of 5-aminolevulinic acid, expression of a heme importer, and addition of the entire heme biosynthesis pathway. While improved heme supply showed promise, fine-tuning of the pathway is necessary as too high intracellular concentrations of heme started inhibiting P450 activity (Hu et al. 2023). In *B. subtilis*, improving heme supply led to a 3-fold increase in hemoglobin titer compared to the wildtype (Wang et al. 2025). Therefore, improving heme biosynthesis in *P. putida* could be evaluated as a strategy to improve the co-expression of the cytochrome P450 and hemoglobin genes. Recently, a non-heme Rieske monooxygenase capable of demethylating guaiacol was discovered and expressed in *P. putida* (Bleem et al. 2022). This could also be used as a model system to investigate the same reaction (guaiacol conversion) but in a non-heme dependent manner.

The persistent negative effect of over-expressing *AHb2* for whole-cell bioconversion of guaiacol in medium supplemented with ALA indicates that the heme supply may not be the only factor to incriminate. Competition for molecular oxygen could arise as both proteins bind oxygen as part of their function, but with different affinities. Although the precise affinity for oxygen of the P450 used in this study is not known, P450s generally have a low oxygen affinity to reduce the likelihood of side-reactions occurring, which could result in the formation of harmful intermediates. Moreover, binding of oxygen is dependent on substrate binding and electron transfer (Lewis and Sheridan 2001). Hemoglobin from *A. thaliana*, on the other hand, has a high affinity, allowing it to bind oxygen effectively at low concentrations. Therefore, hemoglobin is more likely to bind the available oxygen than the cytochrome P450. Consequently, less oxygen would be available for the cytochrome P450. Finding hemoglobin candidates with higher oxygen affinity may be more compatible with cytochrome P450 systems. Furthermore, as previously mentioned, expression of heterologous hemoglobin genes has been associated with changes in respiratory activity. In the present study, however, the hemoglobin-expressing strain exhibited reduced carbon dioxide evolution rates, together with a decrease in respiratory quotient. These observations indicate a shift in respiratory metabolism rather than an overall increase in respiratory activity. Such changes may affect the intracellular redox balance. The NADH-dependent GcoAB system relies on the availability of reducing equivalents, and alterations in the coupling between respiration and carbon metabolism may therefore limit its activity. In addition, co-expression of multiple heterologous proteins may impose a burden on the cellular protein folding and maturation machinery. Such effects could influence enzyme functionality and stability, particularly for complex proteins such as cytochrome P450 systems, and may contribute to the reduced bioconversion performance observed. Future studies should investigate the influence of heterologous hemoglobin on redox balance within the host.

## Conclusions

In this study, the use of hemoglobin as an oxygen carrier was explored in *P. putida*. Two recombinant plant hemoglobin genes were successfully expressed in *P. putida*, which showed a tendency toward improved growth on glucose at low oxygen level, although this effect was not statistically significant. Despite this, expression of hemoglobin in a muconic acid producing strain had a negative impact on growth, guaiacol consumption and muconic acid production under well‑controlled bioreactor conditions, despite non‑limiting oxygen availability, and was accompanied by reduced respiration rates and a lower respiratory quotient. While hemoglobin shows promise as an oxygen carrier to improve performance under oxygen‑limitation, its impact on competing oxygen‑demanding reactions and cellular physiology still needs to be evaluated on a case‑by‑case basis.

## Supplementary Information

Supplementary Material 1.

## Data Availability

The datasets generated during and/or analysed during the current study are available from the corresponding author on reasonable request.
