# Supplementary material for: Hemoglobin expression reduces aerobic bioconversion of guaiacol in Pseudomonas putida despite non-limiting oxygen
Source: AMB Express. 2026 Jun 30;16:96. doi: 10.1186/s13568-026-02089-3 (PMC13590471; doi:10.1186/s13568-026-02089-3)
Supplement: Supplementary file 1 [file 13568_2026_2089_MOESM1_ESM.doc]

Hemoglobin expression reduces aerobic bioconversion of guaiacol in *Pseudomonas putida* despite non-limiting oxygen

**Authors**

Fredrik Lund^#1^, Matilda Dellson^#1^, Nélida Leiva Eriksson^1^, Marie Gorwa-Grauslund^1,2*^, Magnus Carlquist^1^*

**Author affiliations**

*^1^Division of Biotechnology and Applied Microbiology, Department of Process and Life Science Engineering, Lund University, SE-221 00 Lund, Sweden*

*^2^Novonesis, Gammel Venlighedsvej 14, 2970, Hoersholm, Denmark*

***Corresponding authors’ email:**

magnus.carlquist@ple.lth.se

mega@novonesis.com

^#^shared first authorship

**List of content**

**Figure S**1**.** SDS-PAGE analysis of P. putida strains expressing recombinant hemoglobin genes (lanes 2-5), carrying an empty vector (lane 6), expressing *AHb2* and *GcoAB* (lane 8), or expressing *GcoAB* (lane 9). Expression product of recombinant hemoglobin genes forms a thick band with a size of 17 kDa.

**Figure S2.** Cell lysate of strains containing VHb, *AHb2* or *BvHb2*

**Figure S3. (A) Quantification of hemoglobin expression in recombinant strains.** Hb levels as quantified spectrometrically from cell lysate. Data represents the mean of three independent biological replicates. Error bars show the standard deviation. **(B) Visual confirmation of hemoglobin expression in bioreactor cultivation**. Representative image of a bioreactor culture of *P. putida* TMBFL038 expressing *AHb2* from *Arabidopsis thaliana*. The intense reddish coloration of the culture is consistent with functional hemoglobin expression and contrasts with the appearance of control cultures lacking hemoglobin.

**Figure S4**. Growth profile of P. putida strains expressing different recombinant hemoglobin genes and an empty vector (control strain, Hb-) in three different aeration conditions: a) baffled shake flask at 180 rpm, b) unbaffled shake flask at 180 rpm, c) baffled shake flask at 0 rpm. Cultivations were performed in M9 supplemented with 10 g/L glucose and 100 µg/mL streptomycin for plasmid maintenance. Data represent the mean of two biological replicates. Error bars indicate the variability between replicates.

**Figure S5. A)** Partial metabolic pathway for vanillin assimilation in P. putida. Abbreviations: VAN, vanillin; VA, vanillic acid; PCA, protocatechuic acid; 3-ccMA, 3-carboxy-cis,cis muconic acid. **B**) Metabolic pathway for the conversion of guaiacol to cis,cis-muconic acid in P. putida. Overexpressed enzymes/genes are marked in green, native in black. Deleted steps are marked by a red cross. Abbreviations: GUA, guaiacol; CAT, catechol; ccMA, cis,cis-muconic acid

**Table S1.** List of codon-optimized gene sequences used in this study.

**Table S2.** List of oligos used in the study. The ribsome binding site (RBS) is in bold; restriction sites are underlined.

**Table S3.** List of hemoglobin proteins used in the phylogenetic analysis.

**Figure S**2**.** SDS-PAGE analysis of P. putida strains expressing recombinant hemoglobin genes (lanes 2-5), carrying an empty vector (lane 6), expressing *AHb2* and *GcoAB* (lane 8), or expressing *GcoAB* (lane 9). Expression product of recombinant hemoglobin genes forms a thick band with a size of 17 kDa.

**Figure S2.** Cell lysate of strains containing VHb, *AHb2* or *BvHb2*

**Figure S3. (A) Quantification of hemoglobin expression in recombinant strains.** Hb levels as quantified spectrometrically from cell lysate. Data represents the mean of three independent biological replicates. Error bars show the standard deviation. **(B) Visual confirmation of hemoglobin expression in bioreactor cultivation**. Representative image of a bioreactor culture in M9+ALA of *P. putida* TMBFL038 expressing *AHb2* from *Arabidopsis thaliana*. The intense reddish coloration of the culture is consistent with functional hemoglobin expression and contrasts with the appearance of control cultures lacking hemoglobin.

**Figure S4**. Growth profile of P. putida strains expressing different recombinant hemoglobin genes and an empty vector (control strain, Hb-) in three different aeration conditions: a) baffled shake flask at 180 rpm, b) unbaffled shake flask at 180 rpm, c) baffled shake flask at 0 rpm. Cultivations were performed in M9 supplemented with 10 g/L glucose and 100 µg/mL streptomycin for plasmid maintenance. Data represent the mean of two biological replicates. Error bars indicate the variability between replicates.

**Figure S5. (A)** Partial metabolic pathway for vanillin assimilation in P. putida. Abbreviations: VAN, vanillin; VA, vanillic acid; PCA, protocatechuic acid; 3-ccMA, 3-carboxy-cis,cis muconic acid. **(B)** Metabolic pathway for the conversion of guaiacol to cis,cis-muconic acid in P. putida. Overexpressed enzymes/genes are marked in green, native in black. Deleted steps are marked by a red cross. Abbreviations: GUA, guaiacol; CAT, catechol; ccMA, cis,cis-muconic acid

**Table S1.** List of codon-optimized gene sequences used in this study.

**GeneCodon-optimized sequence***AHb2*ATGGGAGAGATTGGGTTTACAGAGAAGCAAGAAGCTTTGGTGAAGGAATCGTGGGAGATACTGAAACAAGACATCCCCAAATACAGCCTTCACTTCTTCTCACAGATACTGGAGATAGCACCAGCAGCAAAAGGCTTGTTCTCTTTCCTAAGAGACTCAGATGAAGTCCCTCACAACAATCCTAAACTCAAAGCTCATGCTGTTAAAGTCTTCAAGATGACATGTGAAACAGCTATACAGCTGAGGGAGGAAGGAAAGGTGGTAGTGGCTGACACAACCCTCCAATATTTAGGCTCAATTCATCTCAAAAGCGGCGTTATTGACCCTCACTTCGAGGTGGTGAAAGAAGCTTTGCTAAGGACATTGAAAGAGGGGTTGGGGGAGAAATACAATGAAGAAGTGGAAGGTGCTTGGTCTCAAGCTTATGATCACTTGGCTTTAGCCATCAAGACCGAGATGAAACAAGAAGAGTCATAA*BvHb2*ATGACTTTTACAGAGAAAGATGAAGCTTTGGTAAAAGAATCATGGGATATAATGAAGCAAAATATCCCAGAATACAGCCTTCGGTTTTTCTCCATAATATTGGAAATTGCTCCAGCAGCCAAAAATATGTTCTCATTTTTAAGGGATTCAGAGGAAGTTCCACAGAATAATCCCAAGCTGAAAGCTCATGCAATCAAGGTTTTTAAAATGACATGTGAATCAGCCATTCAACTTCGAGAAAAAGGTGAAGTGGTTGTAGGAGAGACTACCCTTAAATATTTGGGAGCTATCCATTTGAAGAATGGAGTGATTGATCCCCATTTTGAGGTTGTGAAACAAGCATTATTGAGAACCATAGAAGAAGCAAGTGGTGACAAATGGAGTGAAGAATTGAAATGTGCTTGGAGTGTTGCCTATGATCACTTAGCTGCAGCCATCAAAGCTGAGATGAAGGAATAG

**Table S2.** List of oligos used in the study. The ribosome binding site (RBS) is in bold; restriction sites are underlined.

**Primer nameSequencePurpose**FL_AtHB_rev_RBS_KpnIAATGGTACCA**GGAGGAAAAACCC**ATGGGAGAGATTGGGTTTACAGAGAConstruction of pFL030

FL_AtHb_fw_BamHIAATGGATCCTTATGACTCTTCTTGTTTCATCTCGGTCConstruction of pFL030

FL_BvHb_RBS_KpnI_fwAATGGTACCAGGAGGAAAAACCCATGACTTTTACAGAGAAAGATGAAGCTTConstruction of pFL034

FL_BvHb_rev_BamHIAATGGATCCCTATTCCTTCATCTCAGCTTTGATGGConstruction of pFL034

FL_AtHb_RBS_XmaJIAATCCTAGGAGGAG GAAAAACCCATGGG AGAGATTGGGTTTAC AGAGAConstruction of pFL038

FL_AtHb_rev_SacIAATGAGCTCTTATGA CTCTTCTTGTTTCAT CTCGGTCConstruction of pFL038

**Table S3.** List of hemoglobin proteins used in the phylogenetic analysis.

**NameOrganism of OriginSequenceDatabase IDReference of use**VHb*Vitreoscilla stercoraria*MLDQQTINIIKATVPVLKEHGVTITTTFYKNLFAKHPEVRPLFDMGRQESLEQPKALAMTVLAAAQNIENLPAILPAVKKIAVKHCQAGVAAAHYPIVGQELLGAIKEVLGDAATDDILDAWGKAYGVIADVFIQVEADLYAQAVE

Uniprot: **P04252**

(Khosla & Bailey, 1988)*AHb2Arabidopsis thaliana*MGEIGFTEKQEALVKESWEILKQDIPKYSLHFFSQILEIAPAAKGLFSFLRDSDEVPHNNPKLKAHAVKVFKMTCETAIQLREEGKVVVADTTLQYLGSIHLKSGVIDPHFEVVKEALLRTLKEGLGEKYNEEVEGAWSQAYDHLALAIKTEMKQEES

**Uniprot ID: O24521**This study*BvHb2Beta vulgaris*MTFTEKDEALVKESWDIMKQNIPEYSLRFFSIILEIAPAAKNMFSFLRDSEEVPQNNPKLKAHAIKVFKMTCESAIQLREKGEVVVGETTLKYLGAIHLKNGVIDPHFEVVKQALLRTIEEASGDKWSEELKCAWSVAYDHLAAAIKAEMKE

NCBI Reference Sequence: NP_001290022.1This studyAmHb*Arenicola marina*MKVLIVLMACLAYVAADCGPLQRLKVKHQWVQVYSGHGYEREAFGREVFLEMYNQAPKAKDLFTRVRGENVFSPEFGAHMVRVLGGLDMCIALLSDDTVLNAQLAHLSTQHKDRGIPNEYFDVMKVALMKVVPGHVSHFDFDAWSACYDVIANGIKH

GenBank: CAJ32741.1(Harnois et al., 2009)OsHb*Oryza sativa*MALVEDNNAVAVSFSEEQEALVLKSWAILKKDSANIALRFFLKIFEVAPSASQMFSFLRNSDVPLEKNPKLKTHAMSVFVMTCEAAAQLRKAGKVTVRDTTLKRLGATHLKYGVGDAHFEVVKFALLDTIKEEVPADMWSPAMKSAWSEAYDHLVAAIKQEMKPAE

NCBI Reference Sequence: NP_001389049.1(Giordano et al., 2010)HvHb*Hordeum vulgare*MSAAEGAVVFSEEKEALVLKSWAIMKKDSANLGLRFFLKIFEIAPSARQMFPFLRDSDVPLETNPKLKTHAVSVFVMTCEAAAQLRKAGKITVRETTLKRLGGTHLKYGVADGHFEVTRFALLETIKEALPADMWGPEMRNAWGEAYDQLVAAIKQEMKPAE

NCBI Reference Sequence: XP_044979241.1(Duff et al., 1997)LpHbPhaicoides pectinatusMSLSAAQKDNVKSSWAKASAAWGTAGPEFFMALFDAHDDVFAKFSGLFKGAAKGTVKNTPEMAAQAQSFKGLVSNWVDNLDNAGALEGQCKTFAANHKARGISAGQLEAAFKVLAGFMKSYGGDEGAWTAVAGALMGMIRPNM

UniProt ID: P41260.4(León et al., 2004)PmHbScHb*Saccharomyces cerevisiae*MLAEKTRSIIKATVPVLEQQGTVITRTFYKNMLTEHTELLNIFNRTNQKVGAQPNALATTVLAAAKNIDDLSVLMDHVKQIGHKHRALQIKPEHYPIVGEYLLKAIKEVLGDAATPEIINAWGEAYQAIADIFITVEKKMYEEALWPGWKPFDITAKEYVASDIVEFTVKPKFGSGIELESLPITPGQYITVNTHPIRQENQYDALRHYSLCSASTKNGLRFAVKMEAARENFPAGLVSEYLHKDAKVGDEIKLSAPAGDFAINKELIHQNEVPLVLLSSGVGVTPLLAMLEEQVKCNPNRPIYWIQSSYDEKTQAFKKHVDELLAECANVDKIIVHTDTEPLINAAFLKEKSPAHADVYTCGSLAFMQAMIGHLKELEHRDDMIHYEPFGPKMSTVQV

NCBI Reference Sequence: NP_011750.1

(Buisson & Labbe-Bois, 1998)SiHbAAnadara inaequivalvisMVADAVAKVCGSEAIKANLRRSWGVLSADIEATGLMLMSNLFTLRPDTKTYFTRLGDVQKGKANSKLRGHAITLTYALNNFVDSLDDPSRLKCVVEKFAVNHINRKISGDAFGAIVEPMKETLKARMGNYYSDDVAGAWAALVGVVQAAL

UniProt ID: P14821.2SiHbBAnadara inaequivalvis

SKVAELANAVVSNADQKDLLRMSWGVLSVDMEGTGLMLMANLFKTSPSAKGKFARLGDVSAGKDNSKLRGHSITLMYALQNFVDALDDVERLKCVVEKFAVNHINRQISADEFGEIVGPLRQTLKARMGNYFDEDTVSAWASLVAVVQASL

UniProt ID: P14822.1

SsHb*Synechocystis sp.* MSTLYEKLGGTTAVDLAVDKFYERVLQDDRIKHFFADVDMAKQRAHQKAFLTYAFGGTDKYDGRYMREAHKELVENHGLNGEHFDAVAEDLLATLKEMGVPEDLIAEVAAVAGAPAHKRDVLNQ

NCBI Reference Sequence: WP_010872616.1(Scott & Lecomte, 2000)VuHbVigna unguiculataMVAFSDKQEGLVNGAYEAFKADIPKYSVVFYTTILEKAPAAKNLFSFLANGVDATNPKLTGHAEKLFGLVRDSAAQLRASGGVVADAALGAVHSQKAVNDAQFVVVKEALVKTLKEAVGDKWSDELGTAVELAYDELAAAIKKAY

NCBI Reference Sequence: NP_001363033.1(Arredondo-Peter et al., 1997)

**References**

Arredondo-Peter, R., Moran, J. F., Sarath, G., Luan, P., & Klucas, R. V. (1997). Molecular Cloning of the Cowpea Leghemoglobin II Gene and Expression of Its cDNA in *Escherichia coli* (Purification and Characterization of the Recombinant Protein). *Plant Physiology*, *114*(2), 493-500. https://doi.org/10.1104/pp.114.2.493

Buisson, N., & Labbe-Bois, R. (1998). Flavohemoglobin Expression and Function in *Saccharomyces cerevisiae*: NO RELATIONSHIP WITH RESPIRATION AND COMPLEX RESPONSE TO OXIDATIVE STRESS *. *Journal of Biological Chemistry*, *273*(16), 9527-9533. https://doi.org/10.1074/jbc.273.16.9527

Duff, S. M. G., Wittenberg, J. B., & Hill, R. D. (1997). Expression, Purification, and Properties of Recombinant Barley *Hordeum* sp. Hemoglobin: Optical spectra and reactions with gaseous ligands*. *Journal of Biological Chemistry*, *272*(27), 16746-16752. https://doi.org/10.1074/jbc.272.27.16746

Giordano, P. C., Martínez, H. D., Iglesias, A. A., Beccaria, A. J., & Goicoechea, H. C. (2010). Application of response surface methodology and artificial neural networks for optimization of recombinant *Oryza sativa* non-symbiotic hemoglobin 1 production by *Escherichia coli* in medium containing byproduct glycerol. *Bioresource Technology*, *101*(19), 7537-7544. https://doi.org/https://doi.org/10.1016/j.biortech.2010.05.001

Harnois, T., Rousselot, M., Rogniaux, H., & Zal, F. (2009). High-level Production of Recombinant *Arenicola Marina* Globin Chains in *Escherichia Coli*: A New Generation of Blood Substitute. *Artificial Cells, Blood Substitutes, and Biotechnology*, *37*(3), 106-116. https://doi.org/10.1080/10731190902908445

Khosla, C., & Bailey, J. E. (1988). The *Vitreoscilla* hemoglobin gene: Molecular cloning, nucleotide sequence and genetic expression in *Escherichia coli*. *Molecular and General Genetics MGG*, *214*(1), 158-161. https://doi.org/10.1007/BF00340195

León, R. G., Munier-Lehmann, H., Barzu, O., Baudin-Creuza, V., Pietri, R., López-Garriga, J., & Cadilla, C. L. (2004). High-level production of recombinant sulfide-reactive hemoglobin I from *Lucina pectinata* in *Escherichia coli*: High yields of fully functional holoprotein synthesis in the BLi5 *E. coli* strain. *Protein Expression and Purification*, *38*(2), 184-195. https://doi.org/https://doi.org/10.1016/j.pep.2004.08.014

Scott, N. L., & Lecomte, J. T. J. (2000). Cloning, expression, purification, and preliminary characterization of a putative hemoglobin from the cyanobacterium *synechocystis* sp. PCC 6803. *Protein Science*, *9*(3), 587-597. https://doi.org/https://doi.org/10.1110/ps.9.3.587
